# Impact of Point‐of‐Care Ultrasound in Medicalized Prehospital Setting on Diagnostic Workup: A Prospective Observational Cohort Study

**DOI:** 10.1002/jum.70134

**Published:** 2025-11-21

**Authors:** Frederic Balen, Andy Rouze, Xavier Dubucs

**Affiliations:** ^1^ Emergency Department Toulouse University Hospital Toulouse France; ^2^ CERPOP—EQUITY, INSERM Toulouse France; ^3^ Ottawa Hospital Research Institute, The Ottawa Hospital Ottawa Ontario Canada

**Keywords:** diagnosis, emergency medical service, point‐of‐care ultrasound, prehospital, ultrasound

## Abstract

**Objectives:**

The primary objective of this study was to describe the indications for performing point‐of‐care ultrasound (POCUS) in prehospital settings. The secondary objective was to assess its impact on the diagnostic workup when its use was initiated by the emergency physician (EP) dispatched with a mobile intensive care unit (MICU).

**Methods:**

This prospective observational cohort study was conducted within the MICU of Toulouse University Hospital from December 1, 2022, to May 31, 2023. All adult patients managed by the Toulouse MICU for whom the EP performed a POCUS examination were eligible for inclusion. EP was asked to state the diagnostic hypothesis being evaluated and to rate its likelihood before and after POCUS assessment. The hypothesis and the evaluation of the EP before and after POCUS were compared to the final diagnosis at hospital discharge.

**Results:**

Over the 6‐month study period, 83 had a POCUS by a MICU. The indications for performing POCUS were: high‐energy trauma (n = 50 [60%]), chest pain (n = 20 [24%]), dyspnea (n = 9 [11%]), abdominal pain (n = 3 [4%]), and cardiac arrest (n = 1 [1%]). The diagnostic impression was more often consistent with the final diagnosis after POCUS than before (58 [70%] vs. 36 [43%]; *P* < .001). POCUS modified the diagnostic assessment wrongly in 7 (8%) patients and rightly in 28 (34%) patients.

**Conclusion:**

The most frequent indications for prehospital POCUS were high‐energy trauma, chest pain, and dyspnea. POCUS improved the rate of initial diagnostic assessments consistent with the final diagnosis.

**Abbreviations**
eFAST: Extended Focused Assessment with Sonography for Trauma
EP: emergency physician
MICU: mobile intensive care unit
POCUS: point‐of‐care ultrasound

Point‐of‐care ultrasound (POCUS) was initially developed in the 1990s for use in in‐hospital settings, particularly in North America, leading to the publication of dedicated recommendations as early as 2001.^1^ In France, prehospital emergency physicians (EPs) have played a key role in the dissemination of POCUS, which was historically used for the assessment of high‐energy trauma patients through the Extended Focused Assessment with Sonography for Trauma (eFAST) protocol.^2^ Over the past two decades, a variety of additional prehospital applications have emerged, notably in the management of cardiac arrest, acute dyspnea, and for ultrasound‐guided procedures.^3^, ^4^

Few studies have specifically examined the actual use of POCUS by mobile intensive care units (MICUs), despite the fact that prehospital medicalization remains a distinctive feature of the French emergency care system.^5^ In France, advanced life support teams, known as *MICUs* (mobile intensive care units), are staffed by an EP, a nurse, and an ambulance driver. MICUs are dispatched by the emergency call center (SAMU) to manage the most critical patients. In other cases, the call center may dispatch basic life support ambulances staffed by personnel with more limited training compared with paramedics in other systems^5^—notably, they are not trained to perform POCUS, which is a specific competency of MICU teams. An early study by Lapostolle et al addressed this topic,^6^ but it predated the French Society of Emergency Medicine's guidelines recommending that MICUs be equipped with ultrasound devices to enable prehospital POCUS.^7^ Since then, the availability of ultrasound in MICU vehicles has increased significantly, from 28% in 2016 to 69% in 2023.^8^ Consequently, both the use of POCUS and its impact on patient management have likely evolved.

The primary objective of this study was to describe the indications for performing POCUS during MICU interventions. The secondary objective was to assess its impact on the diagnostic workup when its use was initiated by the EP dispatched with the MICU.

## Methods

This prospective observational cohort study was conducted within the MICU of Toulouse University Hospital over a 6‐month period, from December 1, 2022, to May 31, 2023. In 2023, the Toulouse MICUs carried out approximately 13,000 interventions, including 10,000 primary missions. Training in POCUS varies between EP staffing MICUs. It most often consists of a 7‐day course combining theoretical teaching and hands‐on practice on healthy volunteers followed by a 5‐ to 10‐day clinical fellowship. All adult patients managed by the Toulouse MICU for whom the EP performed a POCUS examination were eligible for inclusion. The treating EP recruited patients at the time of the POCUS examination. The study protocol did not alter clinical practice and was registered in the Toulouse University Hospital's registry of non‐interventional studies (CNIL number: 2206723v0; internal reference: RnIPH 2022‐122) and oral consent to participate was collected from the patient or his/her family. We report our results following the STROBE guidelines for cohort studies.

The primary outcome was the clinical indication for performing POCUS. The secondary outcome was the consistency between the prehospital diagnostic assessment and the final in‐hospital diagnosis, both before and after POCUS, with the discharge diagnosis serving as the reference standard. At inclusion, prior to performing POCUS, the EP was asked to state the diagnostic hypothesis being evaluated (e.g., “search for serous effusion via eFAST”) and to rate the likelihood of this hypothesis using a Likert scale from 0 (very unlikely) to 10 (very likely). After the POCUS examination, the EP was again asked to rate the likelihood of the same hypothesis using the same scale. The EP collected those data during the inclusion process. Final diagnoses related to the tested hypotheses were determined retrospectively by the study investigators (FB, AR) based on the patient's hospital records. Diagnostic assessments were then categorized as follows:

- Consistent with the final diagnosis: the hypothesis was confirmed and the EP estimated the hypothesis tested to be likely (≥7), or the hypothesis was refuted and the EP estimated the hypothesis unlikely (≤3).
- Diagnostic uncertainty: the EP estimated the hypothesis tested uncertain (4, 5, or 6 upon 10)
- Not consistent with the final diagnosis: the hypothesis was confirmed but the EP estimated the hypothesis tested unlikely (≤3), or the hypothesis was refuted but the EP estimated the hypothesis tested to be likely (≥7).

Due to the absence of preliminary data in our center, no formal sample size calculation was performed. A 6‐month data collection period was considered appropriate to describe the target population. Statistical analysis was conducted on anonymized data using STATA software (version 16; StataCorp, College Station, TX, USA) by FB. Age is presented as median and interquartile range (m [25th–75th percentile]). Categorical variables are expressed as counts and percentages. Diagnostic consistency before and after POCUS was compared using Fisher's exact test.

## Results

**Figure 1:**
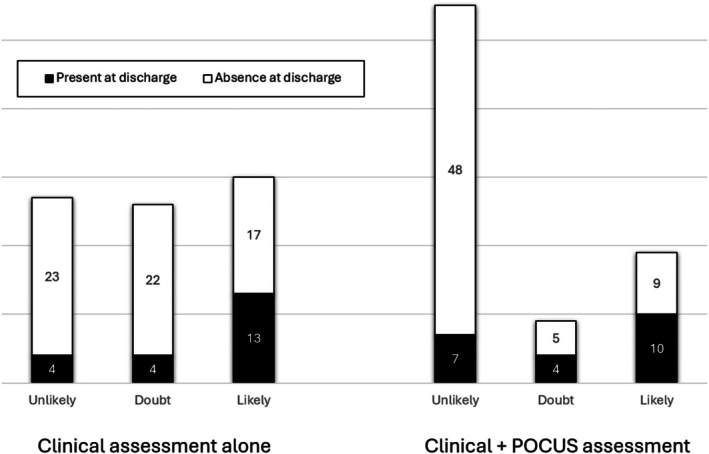
Diagnostic tested assessment before and after POCUS.

Over the 6‐month study period, the Toulouse adult SMUR performed nearly 5000 primary interventions, of which 83 (2%) benefited from a POCUS and were included. Patients' median age was 44 (23–70) years and 62% (n = 51) were male (Table 1). The indications for performing POCUS during MICU interventions were: high‐energy trauma (n = 50 [60%]), chest pain (n = 20 [24%]), dyspnea (n = 9 [11%]), abdominal pain (n = 3 [4%]) and cardiac arrest (n = 1 [1%]). No POCUS was performed for ultrasound guidance. Figure 1 shows hypothesis tested assessment probability before and after POCUS. Table 2 shows the diagnostic assessment consistency before and after POCUS. The diagnostic impression was more often consistent with the final diagnosis after POCUS than before (58 [70%] vs. 36 [43%]; *P* < .001). POCUS did not modify the EP assessment in 48 (58%) patients. However, it did modify the diagnostic assessment, wrongly in 7 (8%) patients and rightly in 28 (34%) patients. Specific analyses were carried out on the following subgroups: POCUS performed for high‐energy trauma (Table 3), chest pain (Table 4), and dyspnea (Table 5). In high‐velocity trauma cases, POCUS significantly improved consistency with the final diagnosis (32 [64%] vs. 18 [36%]; *P* = .022). A similar effect was observed among patients presenting with chest pain (14 [70%] vs. 10 [50%]; *P* = .007). In patients with shortness of breath, however, the positive impact of POCUS was not statistically significant (8 [89%] vs. 5 [56%]; *P* = .444).

**Table 1:** Patient Characteristics, Chief Complaint, and Diagnostic Researched

	Population (n = 83)	Diagnosis Researched Present at Discharge
Age	44 (23–70)	
Male sex	51 (62)	
Traumatology (eFAST)	50 (60)	13 (26)
Chest pain	20 (24)	
Search for aortic dissection	10 (12)	1 (10)
Search for pericardial effusion	4 (5)	0
Search for pulmonary embolism	3 (4)	1 (25)
Search for coronary syndrome	2 (2)	1 (50)
Search for pneumothorax	1 (1)	0
Dyspnea	9 (11)	
Search for acute lung edema	7 (8)	3 (43)
Search for pneumothorax	1 (1)	1 (100)
Search for pulmonary embolism	1 (1)	0
Abdominal pain	3 (4)	
Search for abdominal aortic aneurysm	2 (2)	1 (50)
Pregnancy search	1 (1)	0
Cardiac arrest (prognostic evaluation)	1 (1)	0^a^

^a^ Research of cardiac activity (none), death in prehospital settings.

**Table 2:** Diagnostic Assessment Before and After POCUS in Prehospital Setting

		Clinical Assessment + POCUS				
		Not Consistent With Final Diagnosis	Uncertain	Consistent With Final Diagnosis	Total	*P*
Clinical assessment alone	Not consistent with final diagnosis	11 (13)	2 (2)	8 (10)	21 (25)	<.001
	Uncertain	3 (4)	5 (6)	18 (22)	26 (31)	
	Consistent with final diagnosis	2 (2)	2 (2)	32 (39)	36 (43)	
	Total	16 (19)	9 (11)	58 (70)	83 (100)	

In yellow: POCUS did not change assessment; in green: POCUS improved diagnosis work‐up; in red: POCUS negatively altered diagnosis work‐up.

POCUS, point‐of‐care ultrasound.

**Table 3:** Diagnostic Assessment Before and After eFAST for High Velocity Trauma

		Clinical Assessment + POCUS				
		Not Consistent With Final Diagnosis	Uncertain	Consistent With Final Diagnosis	Total	*P*
Clinical assessment alone	Not consistent with final diagnosis	8 (16)	1 (2)	6 (12)	15 (30)	.022
	Uncertain	3 (6)	3 (6)	11 (22)	17 (34)	
	Consistent with final diagnosis	1 (2)	2 (4)	15 (30)	18 (36)	
	Total	12 (24)	6 (12)	32 (64)	50 (100)	

**Table 4:** Diagnostic Assessment Before and After POCUS for Chest Pain

		Clinical Assessment + POCUS				
		Not Consistent With Final Diagnosis	Uncertain	Consistent With Final Diagnosis	Total	*P*
Clinical assessment alone	Not consistent with final diagnosis	2 (10)	1 (5)	0	3 (15)	.007
	Uncertain	0	2 (10)	5 (25)	7 (35)	
	Consistent with final diagnosis	1 (5)	0	9 (45)	10 (50)	
	Total	3 (15)	3 (15)	14 (70)	20 (100)	

**Table 5:** Diagnostic Assessment Before and After POCUS for Dyspnea

		Clinical Assessment + POCUS				
		Not Consistent With Final Diagnosis	Uncertain	Consistent With Final Diagnosis	Total	*P*
Clinical assessment alone	Not consistent with final diagnosis	1 (11)	0	1 (11)	2 (22)	.444
	Uncertain	0	0	2 (22)	2 (22)	
	Consistent with final diagnosis	0	0	5 (56)	5 (56)	
	Total	1 (11)	0	8 (89)	9 (100)	

## Discussion

The most frequent indications for performing POCUS during MICU interventions in our cohort were high‐energy trauma, chest pain, and dyspnea. When the prehospital EP deemed POCUS indicated, its use was associated with an increased rate of initial diagnostic assessments consistent with the final diagnosis. Lapostolle et al previously reported similar findings, with a high prevalence of prehospital POCUS performed for suspected peritoneal or pleural effusion (47% and 29%, respectively) in a prospective study involving 302 POCUS examinations.^6^ In contrast to our findings, their study included fewer cases of POCUS for pericardial effusion (5%) or other indications (7%). They also reported a diagnostic benefit in 67% of cases, no benefit in 25%, and a negative impact in 8%.^6^ This higher rate of positive diagnostic contribution compared with our results may be explained by methodological differences: while Lapostolle et al considered any shift in diagnostic impression score as a benefit, we only considered categorical changes in diagnostic class (e.g., from “diagnostic uncertainty” [Likert 4–6] to “hypothesis likely” [Likert ≥7]).

A limitation of our study is that EPs were asked to provide a single diagnostic hypothesis motivating the use of POCUS, whereas in clinical reality, multiple hypotheses may coexist—especially in cases of chest pain or dyspnea. Another key limitation is the monocentric and French nature of our study, which may restrict its external validity. The indications for MICU dispatch and their frequency likely influence the type of POCUS performed. In our region, chest pain and cardiovascular complaints account for 43% of primary MICU dispatches, while trauma and dyspnea account for 14% and 8%, respectively. This high frequency of chest pain‐related dispatch likely explains the high number of POCUS performed for this indication in our cohort, despite ongoing debate regarding its diagnostic value in the prehospital setting.

Although data on prehospital POCUS for cardiocirculatory complaints remain limited, several authors have suggested that its use mirrors in‐hospital applications—particularly cardiac and pleuropulmonary ultrasound in patients with chest pain.^9^ While POCUS is not reliable for ruling out acute coronary syndrome, it may help identify alternative causes of chest pain.^10^ Detection of pericardial effusion is a valuable finding suggestive of pericarditis,^11^ and although specific ultrasound signs of aortic dissection exist, its sensitivity is limited.^12^ Nevertheless, both diagnoses—pericarditis and aortic dissection—are rare in MICU‐dispatched chest pain patients.^13^ Moreover, MICU intervention is unlikely to significantly influence the patient's referral to the emergency department in these cases. The use of POCUS for trauma, especially through eFAST, was substantial in our cohort. Although its sensitivity for detecting thoracoabdominal effusion in the prehospital setting is limited, its prognostic value is well documented.^14^ Indeed, the presence of effusion on prehospital eFAST is an independent risk factor for severe hemorrhage and may aid in directing patients toward appropriate in‐hospital resources.^15^ POCUS was infrequently used for dyspnea in our study, likely reflecting the low frequency of MICU dispatch for this symptom. However, previous research has shown that prehospital POCUS performs well in the diagnostic evaluation of dyspnea.^16^, ^17^ Its use in cardiac arrest was also limited in our cohort, although it may provide prognostic information and aid in identifying reversible causes of arrest.^18^, ^19^

## Conclusion

In this cohort, the most frequent indications for prehospital POCUS during MICU interventions were high‐energy trauma, chest pain, and dyspnea. When its use was considered appropriate by the EP, POCUS improved the rate of initial diagnostic assessments consistent with the final diagnosis.

## Data Availability

The data that support the findings of this study are available from the corresponding author upon reasonable request.
